# Professional Values, Perceived Ethical Climate, and Dignity‐Protecting Behaviors Among Intensive Care Nurses: A Moderation Analysis

**DOI:** 10.1155/jonm/6857380

**Published:** 2026-09-25

**Authors:** Merve Tarhan, Pınar Doğan, Rabia Eren

**Affiliations:** ^1^ Nursing, School of Health Sciences, İstanbul Medipol University, Istanbul, Turkey, medipol.edu.tr

**Keywords:** dignity-protecting behavior, ethical climate, intensive care unit, nursing, professional values

## Abstract

**Background:**

In intensive care unit (ICU) patients, diminished consciousness, device dependency, and limited privacy leave dignity preservation largely dependent on nurses’ behavior, which may be shaped by both individual professional values and the unit’s ethical climate.

**Aim:**

To examine the predictive roles of professional values and perceived ethical climate on dignity‐protecting behaviors among ICU nurses and to test whether perceived ethical climate moderates the professional values–dignity‐protecting behavior relationship.

**Methods:**

This descriptive, cross‐sectional study included 165 nurses from the adult ICUs of six private hospitals in Istanbul, Türkiye. Data were collected using the Nurses’ Professional Values Scale, the Intensive Care Unit Dignified Care Scale, and the Hospital Ethical Climate Scale. Regression analyses, Hayes’ PROCESS macro, and simple slope analysis were used to test the moderation model.

**Results:**

Professional values (*β* = 0.521) and perceived ethical climate (*β* = 0.679) significantly predicted dignity‐protecting behaviors, with large effect sizes. The interaction term significantly predicted dignity‐protecting behaviors (*B* = −0.005, *p* < 0.001): The effect of professional values was significant at low and mean levels of ethical climate, but not at high levels.

**Conclusion:**

Professional values and ethical climate independently predicted dignity‐protecting behaviors, and ethical climate moderated this relationship, with professional values mattering most when ethical climate was weak. A strongly perceived ethical climate appears to reduce, rather than amplify, the behavioral influence of professional values, offering a more consistent pathway to safeguarding patient dignity in ICUs.

**Implications for Nursing Management:**

Since professional values mattered most under weak ethical climates, nurse managers should pair efforts to strengthen the ethical work environment with strategies to reinforce nurses’ individual professional values, including transparent decision‐making, sensitivity to patient rights, and support for ethical discussion within ICU teams.

## 1. Introduction

The aging population and rising prevalence of chronic diseases in Türkiye are progressively expanding the demand for intensive care services. Among Organization for Economic Co‐operation and Development (OECD) countries, Türkiye ranks third in the number of adult intensive care beds per 100,000 population, with 38.2 beds, and its bed occupancy rate of 68.9% exceeds the OECD average. In contrast, the number of nurses per 100,000 population in Türkiye remains considerably below the average of these countries, ranking last among them [1]. Accordingly, national legislation mandates the employment of at least one nurse for every two occupied beds in level three intensive care units (ICUs) [2]. However, the existing nursing shortage forces ICU nurses to care for more critically ill patients simultaneously, intensifying workload.

Under heavy workload, nurses’ attention shifts from the patient’s subjective experience toward measurable indicators such as vital signs and device data, creating distance between nurse and patient in technology‐ICU settings [3]. Furthermore, nurses have also been observed to prioritize physical care over patients’ psychosocial needs and participation in their own care, reflecting a strongly task‐oriented approach [4]. This pattern is reportedly amplified in the ICU, where continuous emergency demands, heavy technology reliance, and workload can relegate patients’ individuality to the background [5]. With consciousness often diminished, vital functions device‐dependent, and privacy physically limited, ICU patients largely lose voice and control over their own care [6]. Consequently, once patients lose this control entirely, the preservation of their dignity depends not on their own agency but directly on the nurse’s attitudes and behavior.

## 2. Background

Dignity‐protecting behaviors are defined as attitudes and practices in which the patient’s worth as a human being, autonomy, and privacy are respected. This concept is addressed within two dimensions: absolute dignity, which is inherent to and equally held by every individual simply by virtue of being human, and relative dignity, which is shaped by an individual’s social status, cultural background, and personal values [7]. Yet, acute care patients have been reported to experience dignity loss ranging from mild to severe, with this loss occurring at higher rates in Asian countries compared to Europe [8]. Indeed, in a Chinese ICU sample, nurses were found to largely meet absolute dignity requirements such as privacy while falling short in preserving relative dignity [9]. This result suggests that dignity‐protecting behavior should be considered a multidimensional construct rather than a uniform one. In Türkiye, ICU nurses have been found to hold moderate‐to‐high adherence to principles of dignified death, while more than three‐quarters report having no knowledge of the concept of dignified death [10].

Dignity‐violating behaviors, defined as the absence or reversal of dignity‐protecting behavior, are documented concretely in ICU‐specific qualitative studies. Patients identify procedures performed without consent, vague technical explanations, and care given without eye contact or verbal communication as sources of objectification and dignity loss [11]. Another such behavior is the desensitization nurses develop toward repeated death experiences: over time, dying‐related interventions shift from individualized care to routine tasks, reducing attention during critical moments such as resuscitation [12]. These behaviors are classified into six categories—disrespect for privacy, exclusion from decision‐making, ignoring patients, aggression, stigmatization, and inadequate end‐of‐life care—stemming from both individual attitudes and organizational factors such as inadequate funding and inexperienced staffing [13]. Consistent with this, ICU nurses have been observed to voice commitment to dignity and equality while exhibiting objectifying, paternalistic behavior in practice [14], raising the question of what drives nurses’ behavior and directing attention toward professional values as the foundation of their professional identity.

Professional values are standards of action adopted by members of a profession that guide nurses’ beliefs, attitudes, and behavior [15]. Studies specific to ICU nurses show that these values translate into concrete clinical outcomes: Job security and career choice intention enhance compassion satisfaction and reduce burnout [16], while commitment to professional values predicts caring behavior [17, 18]. This relationship appears particularly consequential in the ICU, where clinical complexity and advanced technology can negatively affect caring behavior [17]. Nonetheless, this relationship is neither linear nor unconditional; a Turkish study found that nurses with high professional values sometimes also reported high moral distress and intention to leave the profession [19], suggesting that strong values alone do not guarantee positive outcomes and may even intensify value‐practice tension in unsupportive organizational contexts. The literature further notes that research linking professional values to behavioral outcomes in critical care remains limited [17]. Together, these results suggest that the translation of professional values into behavior depends on the organizational context in which nurses work.

ICUs are among the most stressful and dynamic hospital units, as critical, irreversible decisions are made under uncertainty and time pressure, with nurses continually facing complex ethical issues [20, 21]. The most common ethical problems in this setting involve autonomy‐limiting practices such as inadequate informed consent, privacy violations, disproportionate treatment, deficient end‐of‐life care, and physical restraint [22, 23]. How nurses handle these problems, however, appears linked less to individual values than to the organizational context: restraint decisions, for instance, are shaped more by perceived colleague and managerial support than by nurses’ own values [23]. Situations without a single right answer, such as withdrawing or continuing life support, futile care, and family‐patient disagreements over decision‐making, represent some of the most visible tests of ethical behavior [21, 24], and nurses’ inability to provide adequate emotional support or enable a dignified death is associated with the highest levels of moral distress [25, 26]. Taken together, this evidence highlights ethical climate as an important contextual factor associated with nurses’ day‐to‐day clinical decisions and behavior.

Ethical climate refers to perceptions within an institution about which behaviors are appropriate and how ethical issues are resolved, shaped through nurses’ relationships with their unit, managers, colleagues, and physicians. Although ethical climate represents an organizational phenomenon, it can be assessed through individual perceptions of the ethical characteristics of their work environment [27]. A negative ethical climate in the ICU has concrete organizational consequences: Turnover intention rises in units with weak mutual respect and limited interdisciplinary dialog [28], and nurses perceive less involvement and respect than physicians [29]. A positive climate strengthens moral resilience through moral courage, though moral sensitivity plays no significant role [30], and while it consistently improves performance [31], it does not alone protect against burnout when workload is high [32]. Ethical climate is thus associated with nurses’ coping capacity, moral resilience, collaboration, turnover intention, and performance. However, evidence on its relationship with individual professional values remains mixed: Its weak association with nurses’ ethical values [23] and partial explanatory power for moral resilience [30] suggest that perceived ethical climate and professional values may represent distinct but complementary influences on nurses’ behavior. Accordingly, the extent to which professional values translate into dignity‐protecting behavior may vary depending on how nurses perceive the ethical characteristics of their work environment.

**FIGURE 1:**
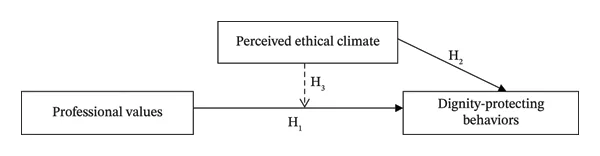
Theoretical model.

This study aims to examine the moderating role of perceived ethical climate in the relationship between professional values and dignity‐protecting behaviors among intensive care nurses. The proposed conceptual model is primarily grounded in trait activation theory, which posits that individual traits translate into behavior only when matched by appropriate situational cues. According to this theory, a trait is a latent disposition that remains constant within the individual but is expressed behaviorally only under trait‐relevant situational conditions; strong and consistent situational cues facilitate this expression, whereas weak or ambiguous cues constrain it [33]. This expectation, however, stands in direct contrast to situational strength theory, which posits that strong situational cues, characterized by clarity, consistency, and unambiguous normative expectations, restrict rather than facilitate the expression of individual differences, thereby homogenizing behavior across individuals [34]. Applied to the present context, these two theoretical perspectives generate opposing predictions: a strong and clearly perceived ethical climate may either strengthen the translation of professional values into dignity‐protecting behavior, as predicted by trait activation theory, or weaken it, as predicted by situational strength theory (Figure 1). The present study was designed to test these competing predictions empirically.

Within this framework, professional values are positioned as an internalized trait underlying nurses’ professional identity, encompassing principles such as respect for human dignity, autonomy, and justice. In the present study, perceived ethical climate was operationalized and analyzed at the individual level, reflecting nurses’ subjective evaluation of the ethical characteristics of the work environment rather than an aggregated unit‐level construct. Dignity‐protecting behaviors are considered the behavioral outcome, encompassing professional practices that safeguard patients’ privacy, autonomy, and human dignity in the ICU setting. Building on these competing theoretical perspectives, perceived ethical climate is expected to moderate the relationship between professional values and dignity‐protecting behaviors, with the direction of this moderating effect tested empirically against the two opposing predictions outlined above. Three core hypotheses were developed within the proposed conceptual model:

• Professional values significantly predict dignity‐protecting behaviors (H_1_).
• Perceived ethical climate significantly predicts dignity‐protecting behaviors (H_2_).
• Consistent with trait activation theory, perceived ethical climate strengthens the relationship between professional values and dignity‐protecting behaviors (H_3a_).
• Consistent with situational strength theory, perceived ethical climate weakens the relationship between professional values and dignity‐protecting behaviors (H_3b_).

## 3. Method

### 3.1. Study Design and Setting

A descriptive, cross‐sectional study was conducted in Istanbul, Türkiye. The study setting comprised the ICUs of six private hospitals affiliated with a single foundation, selected based on their shared institutional culture, management structure, and nursing care standards, thus providing a relatively homogeneous context for examining the study variables. This study was reported in accordance with the STROBE Statement Checklist for cross‐sectional studies.

### 3.2. Study Population and Sample

The inclusion criteria were (a) providing direct patient care in the adult ICU; (b) having at least 6 months of professional experience as an ICU nurse; and (c) voluntarily agreeing to participate in the study. The exclusion criteria were serving in an administrative position without direct patient care responsibilities, being on leave during the data collection period, and having incomplete questionnaire data.

Of the 220 nurses employed in the adult ICUs of the six hospitals, 182 met the inclusion criteria based on employment records at the start of the study period. Of these eligible nurses, 5 subsequently went on leave before questionnaire distribution could be completed, 8 declined to participate, and 4 submitted incomplete questionnaires, resulting in the exclusion of 17 nurses from the final sample. Consequently, the final sample consisted of 165 nurses, corresponding to a participation rate of 90.7% among eligible nurses (165/182) and an overall inclusion rate of 75.0% relative to the total ICU nursing staff (165/220).

An a priori sample size calculation was conducted using G∗Power 3.1.3 for linear multiple regression, assuming a medium effect size (*f* ^2^ *= *0.15), *α* = 0.05, statistical power of 0.80, and three predictors, yielding a minimum required sample size of 77 participants. Given that the primary analysis involved moderation, a sensitivity analysis was additionally conducted for the incremental variance explained by the interaction term (fixed model, *R* ^2^ increase; one tested predictor and three total predictors). With the final sample of 165 participants and *α* = 0.05, the study had 80% power to detect an interaction effect of approximately *f* ^2^ *= *0.048.

### 3.3. Instruments

The self‐administered questionnaire used in this study consisted of four parts: demographic data, the Nurses’ Professional Values Scale, the Intensive Care Unit Dignified Care Scale, and the Hospital Ethical Climate Scale.

Demographic data included age, gender, professional experience, and length of employment in the ICU.

Professional values were measured using the Nurses’ Professional Values Scale, developed by Weis and Schank (2000) to identify the professional values held by nurses based on the ethical code of the American Nurses Association. The Turkish validity and reliability study of the scale was conducted by Şahin Orak and Alpar (2012). This scale comprises 31 items, and each item is rated as 1 = *not important*, 2 = *somewhat important*, 3 = *important*, 4 = *very important*, and 5 = *most important*. All items on the scale are positively worded, and there are no reverse‐scored items. Total scores on the scale range from 31 to 155. Higher scores on the scale indicate that nurses place greater importance on professional values and ethical issues. Cronbach’s *α* coefficient was 0.95 for the adapted version [35].

Dignity‐protecting behaviors were measured using the Intensive Care Unit Dignified Care Scale, developed by Liang et al. (2022) to determine whether ICU nurses exhibit dignity‐protecting care attitudes and behaviors toward patients. The Turkish validity and reliability study of the scale was conducted by Bulut and Şengül (2024). This scale comprises 17 items, and each item is rated as 1 = *never*, 2 = *rarely*, 3 = *occasionally*, 4 = *often*, and 5 = *always*. All items on the scale are positively worded, and there are no reverse‐scored items. Total scores on the scale range from 17 to 85. ICU nurses’ dignity‐protecting care behaviors are evaluated based on a standardized scoring method, calculated using the formula (total score − minimum score)/(maximum score − minimum score) × 100. According to this method, a standardized score of ≥ 80 indicates good behavior, 60–79 indicates moderate behavior, and < 60 indicates poor behavior. Higher scores on the scale indicate that ICU nurses provide a higher level of dignity‐protecting care to patients. Cronbach’s *α* coefficient was 0.89 for the adapted version [36].

Perceived ethical climate was measured using the Hospital Ethical Climate Scale, developed by Olson (1995), which was adapted to Turkish by Bahçecik and Öztürk (2003). This scale contains 26 items, and all items on the Likert‐type scale are positively worded and are scored on a 5‐point scale ranging from 1 to 5: *never true*, *rarely true*, *occasionally true*, *usually true*, and *always true*. Total scores for the scale and its subscales are obtained by summing item scores, with possible total scores ranging from 26 to 130. Higher scores indicate higher levels of perceived ethical climate. Cronbach’s *α* coefficient was 0.89 for the adapted version [27].

### 3.4. Data Collection

Data were collected between April and May 2026 using a paper‐and‐pencil, self‐administered questionnaire. The researchers visited each of the six participating hospitals and personally distributed the questionnaires to ICU nurses who met the inclusion criteria. Prior to distribution, the purpose and content of the study were explained to the nurses, and it was emphasized that participation was entirely voluntary. Nurses who agreed to participate completed the anonymous questionnaire in a quiet area of the unit during breaks or between shifts, which took approximately 10–13 min. Completed questionnaires were sealed in individual envelopes and collected by the researchers immediately afterward to ensure confidentiality and minimize missing data.

### 3.5. Data Analysis

Data analysis was conducted using SPSS 21.0 (IBM Corp., Armonk, NY, USA) and Hayes’ (2013) PROCESS macro. The significance level was set at *p* < 0.05. Descriptive statistics, including numbers, percentages, means, and standard deviations, were used to summarize nurses’ individual characteristics. Scale and subscale scores were presented as means and standard deviations, and internal consistency was assessed using Cronbach’s alpha coefficient based on the current sample. The normality of scale and subscale scores was tested through skewness and kurtosis coefficients; as these coefficients fell within the ± 2 range, the assumption of normal distribution was considered satisfied. Pearson correlation analysis was used to examine the relationships among scale scores. Common method bias was assessed using Harman’s single‐factor test.

Prior to the main regression analyses, the associations between demographic data and dignity‐protecting behavior scores were examined using the independent‐samples *t*‐test. The predictive effects of professional values and perceived ethical climate on dignity‐protecting behaviors (H_1_, H_2_) were tested separately using simple linear regression analysis. The competing moderation predictions (H_3a_, H_3b_) were tested jointly using Hayes’ (2013) PROCESS macro (Model 1), with the sign of the interaction coefficient distinguishing between the two: A positive coefficient would support H_3a_ and a negative coefficient would support H_3b_. Prior to the analysis, scores for professional values and perceived ethical climate were mean‐centered to reduce multicollinearity, and an interaction term was computed as the product of these two variables. The analysis first entered the main effects on dignity‐protecting behaviors, followed by the interaction term to test for the presence of a moderating effect. A significant increase in explained variance upon entry of the interaction term (Δ*R* ^2^, *p* < 0.05), along with a significant regression coefficient for the interaction term, was taken as evidence of a moderating effect. The magnitude of the moderating effect was evaluated using Cohen’s *f* ^2^ coefficient, calculated from Δ*R* ^2^; *f* ^2^ values of 0.02, 0.15, and 0.35, which were considered to represent small, medium, and large effect sizes, respectively [37]. When a significant moderating effect was found, simple slope analyses were conducted to examine the relationship between professional values and dignity‐protecting behaviors at low, mean, and high levels (16th, 50th, and 84th percentiles) of perceived ethical climate [38].

### 3.6. Ethical Considerations

Prior to data collection, ethical approval was obtained from the İstanbul Medipol University Ethics Committee (Decision No: 467; Date: 05.03.2026), and institutional permission was granted by the Nursing Services Directorate of the participating hospitals. The study was conducted in accordance with the ethical principles set forth in the Declaration of Helsinki. All participants were informed in detail about the purpose, procedures, and voluntary nature of the study, and written informed consent was obtained prior to participation. Participants were assured that they could withdraw from the study at any point without providing justification and without incurring any adverse consequences. To ensure participant confidentiality, no personally identifying information was collected, and all data obtained were used exclusively for the purposes of this research.

## 4. Results

Table 1 presents the personal characteristics of the nurses. The mean age was 28.4 years (SD = 5.25; range 21–47), and 56.4% of participants were 27 years or younger; the majority were female. Nearly half of the nurses (49.1%) had more than 4 years of professional experience, while 43% had more than 3 years of ICU experience. Nurses predominantly worked night shifts (83%).

**TABLE 1:** Personal characteristics of nurses.

Personal characteristics	Number	Percentage
*Age groups*		
≤ 27 years	93	56.4
> 27 years	72	43.6

*Gender*		
Female	107	64.8
Male	58	35.2

*Professional experience (years)*		
≤ 4 years	84	50.9
> 4 years	81	49.1

*ICU experience (years)*		
≤ 3 years	94	57.0
> 3 years	71	43.0

*Working shift*		
Day	28	17.0
Night	137	83.0

None of the demographic and professional characteristics examined showed a statistically significant association with dignity‐protecting behavior scores (*p* > 0.05). Accordingly, these variables were not included as covariates in the subsequent regression models, and the analysis focused on the theoretically driven predictors specified in the conceptual model.

Table 2 presents the descriptive statistics and correlation analysis for the study variables. Nurses reported high professional values, with a mean score of 124.19 ± 21.30 out of a possible 155. Dignity‐protecting behaviors were also high, with a standardized mean score of 88.28 ± 13.52 (out of 100). Similarly, perceived ethical climate was rated as high, with a mean score of 111.93 ± 16.73 out of a possible 130. All scales demonstrated acceptable reliability in the current sample (Cronbach’s *α* = 0.95–0.97).

**TABLE 2:** Descriptive statistics and correlation analysis.

Variables	Mean ± SD	Cronbach’s alpha	1	2	3
1. Professional values	124.19 ± 21.30	0.97	1		
2. Dignity‐protecting behaviors	88.28 ± 13.52	0.95	0.521^∗^	1	
3. Perceived ethical climate	111.93 ± 16.73	0.96	0.479^∗^	0.679^∗^	1

^∗^Correlation is significant at the 0.001 level.

Professional values were positively correlated with dignity‐protecting behaviors (*r* = 0.521, *p* < 0.001) and perceived ethical climate (*r* = 0.479, *p* < 0.001). Dignity‐protecting behaviors were also significantly and positively correlated with perceived ethical climate (*r* = 0.679, *p* < 0.001).

As presented in Table 3, professional values significantly and positively predicted dignity‐protecting behaviors (*B* = 0.225, *β* = 0.521, *t* = 7.790, *p* < 0.001, 95% CI [0.168, 0.282]), explaining 27.1% of the variance in dignity‐protecting behaviors (*R* ^2^ *= *0.271, *F* (1,163) = 60.690, *p* < 0.001). The effect size was large (*f* ^2^ = 0.372), supporting H_1_. Similarly, perceived ethical climate significantly and positively predicted dignity‐protecting behaviors (*B* = 0.373, *β* = 0.679, *t* = 11.798, *p* < 0.001, 95% CI [0.311, 0.435]), accounting for 46.1% of the variance in dignity‐protecting behaviors (*R* ^2^ = 0.461, *F* (1,163) = 139.182, *p* < 0.001). This effect was also large (*f* ^2^ *= *0.855), supporting H_2_.

**TABLE 3:** Regression analysis of the study.

Hypothesis	Dependent variable	Independent variable	*B*	*β*	*t*	*p*	95% CI		*R* ^2^	*F*	*p*	*f* ^2^	Effect size
							Lower	Upper					
H_1_	Dignity‐protecting behaviors	Professional values	0.225	0.521	7.790	0.000	0.168	0.282	0.271	60.690	*p* < 0.001	0.372	Large
H_2_	Dignity‐protecting behaviors	Perceived ethical climate	0.373	0.679	11.798	0.000	0.311	0.435	0.461	139.182	*p* < 0.001	0.855	Large

*Note:* B, unstandardized coefficients; *β*, standardized coefficients.

Abbreviation: CI, confidence interval.

The results of the moderation analysis are presented in Table 4. The overall model was significant (*R* ^2^ = 0.563, *F* (3, 161) = 69.057, *p* < 0.001). The interaction term was significant and negative (*B* = −0.005, SE = 0.001, *t* = −4.389, *p* < 0.001, 95% CI [−0.008, −0.003]), indicating that the effect of professional values on dignity‐protecting behaviors was moderated by perceived ethical climate, consistent with H_3b_ rather than H_3a_. Its addition significantly increased the explained variance (Δ*R* ^2^ *= *0.052, *F* = 19.264, *p* < 0.001), corresponding to a small‐to‐medium effect size (*f* ^2^ = 0.120). Simple slope analysis showed that the effect of professional values on dignity‐protecting behaviors was significant at low (16^th^ percentile; effect = 0.203, SE = 0.033, *t* = 6.096, *p* < 0.001) and mean levels of perceived ethical climate (50^th^ percentile; effect = 0.093, SE = 0.026, *t* = 3.569, *p* = 0.001), but not significant at high levels of perceived ethical climate (84^th^ percentile; effect = 0.017, SE = 0.033, *t* = 0.514, *p* = 0.608).

**TABLE 4:** The moderating role of perceived ethical climate.

Hypothesis	Variables	*B*	SE	*t*	*p*	95% CI		*R* ^2^	*F*	*p*	Δ*R* ^2^	*f* ^2^	Effect size
						Lower	Upper						
H_3_	Professional values	0.718	0.141	5.095	0.000	0.440	0.996	0.563	69.057	*p* < 0.001	0.052	0.120	Medium
	Perceived ethical climate	0.908	0.141	6.442	0.000	0.630	1.186						
	Interaction	−0.005	0.001	−4.389	0.000	−0.008	−0.003						

*Note: B*, unstandardized coefficients.

Abbreviations: CI, confidence interval; SE, standardized error.

## 5. Discussion

This study examined the moderating role of perceived ethical climate in the relationship between professional values and dignity‐protecting behaviors among ICU nurses. Results showed that both variables significantly predicted dignity‐protecting behaviors, with perceived ethical climate showing a stronger association with dignity‐protecting behaviors than professional values in the separate regression models. Moreover, perceived ethical climate significantly moderated this relationship, indicating that the translation of professional values into behavior varies according to nurses’ perceptions of the ethical characteristics of their work environment.

In this study, professional values were found to significantly and positively predict dignity‐protecting behaviors with a large effect size, supporting H_1_. Qualitative research offers results that conceptually converge with this relationship based on nurses’ own experiences. In a study examining safe care in intensive care, adherence to professional ethical principles is defined as a core component of safe care [39]. In another study examining the professional identity of ICU nurses, nurses report that professional ethics defines the boundaries of their own behavior [40]. Similarly, in a study conducted in Sweden, nurses state that, recognizing the patient’s strong dependency on staff during critical illness, they take on the responsibility of protecting the patient and preserving their integrity [41]. Quantitative research also statistically supports this relationship. A positive, moderate, and significant relationship has been reported between ICU nurses’ professional values and caring behaviors (*r* = 0.429; *p* = 0.001) [18], and professional values have been reported to significantly predict caring behaviors (*β* = 0.287, *p* < 0.001) [17]. In a study conducted with nurses in China, professional competence and professional self‐awareness are emphasized as being among the strongest predictors of dignity‐protecting behaviors [42]. A study conducted in Türkiye reports that nurses’ professional values significantly and positively affect their moral sensitivity and that increased moral sensitivity, in turn, significantly reduces unmet nursing care [43]. Accordingly, the relationship between professional values and dignity‐protecting behaviors appears to emerge consistently across different cultural contexts and through different mechanisms.

While this result is consistent with the literature in terms of the direction of the effect, it differs markedly in terms of the strength of the effect. Two possible explanations account for this difference. First, the scope of the measurement instrument may play a role: The Intensive Care Unit Dignified Care Scale used in the present study measures a narrow construct focused on dignity‐protecting behaviors specific to the intensive care context, whereas the caring behavior scales used in previous research encompass broader and more heterogeneous dimensions [17, 18]. A context‐specific and narrowly scoped measurement instrument would be expected to show a stronger relationship with a specific predictor such as professional values. Second, the distinct context of intensive care itself may contribute to this difference: In ICUs, where patients’ capacity for verbal communication and bodily autonomy is substantially reduced due to sedation, intubation, and invasive procedures, nurses must sustain dignity‐protecting behaviors largely through touch and nonverbal communication [44]. Under these conditions, dignity‐protecting behaviors are reduced to highly specific and observable actions; this may partly explain why an ICU‐specific scale captures this narrow set of behaviors more sensitively than general scales, thereby yielding a higher effect coefficient.

In the present study, perceived ethical climate was also found to significantly and positively predict dignity‐protecting behaviors with a large effect size, supporting H_2_. This result conceptually converges with results obtained from nurses’ experiences in qualitative research. Arman et al. (2025) and Kitila et al. (2026) found ethical commitment and organizational support to be among the core determinants of dignified care [11, 45], whereas Bidabadi et al. (2019) demonstrated the relationship in the reverse direction, showing that a reductionist and paternalistic organizational culture leads to dignity violations, and thus that the absence of a positive ethical climate weakens dignity‐protecting behaviors [14]. Consistent with the present study’s quantitative results, these results support the relationship between ethical climate and dignity‐protecting behaviors at the qualitative level. ICUs are environments in which ethical dilemmas are intensely experienced due to factors such as life‐threatening clinical conditions, limited patient autonomy resulting from sedation and altered consciousness, end‐of‐life decision‐making processes, and the use of advanced technology [5, 46]. Since this structurally intense ethical environment may heighten ICU nurses’ sensitivity to organizational ethical climate, the high effect size observed in the present study can be contextually explained.

While the relationship between professional values and behavioral outcomes has been relatively frequently studied in the literature, the relationship between ethical climate and behavioral outcomes has been examined less often. In one of this limited number of studies, a strong relationship is reported between ethical climate and employee performance among general hospital nurses in Türkiye (*r* = 0.635, *p* < 0.001) [31], while a study conducted with general hospital nurses in Iran reported that ethical climate explained caring behaviors significantly but to a limited extent (*β* = 0.33, *R* ^2^ = 0.09) [47]. In a study specific to intensive care, ethical climate showed a moderate correlation with patient privacy (*r* = 0.54) and, together with nurses’ spiritual well‐being, explained 40% of the variance in patient privacy (*R* ^2^ = 0.40) [48]. All of these studies are consistent with the present study in terms of the direction of the effect; however, the relationship observed in the present study (*β* = 0.679) is markedly stronger. This difference can be explained partly by the scale‐specificity rationale discussed under H_1_ and partly by differences in sample and clinical context: Most of the compared studies were conducted with general hospital nurses, whereas the present study was conducted in the intensive care context, where the ethical burden is relatively high; therefore, these differences should be taken into account when directly comparing effect sizes. It is also noteworthy that perceived ethical climate showed a stronger association with dignity‐protecting behaviors (*β* = 0.679) than professional values (*β* = 0.521) in the separate regression models. While professional values represent an individual characteristic, perceived ethical climate reflects nurses’ appraisal of the ethical characteristics of their work environment. The stronger association observed for perceived ethical climate suggests that contextual perceptions may be more closely associated with dignity‐protecting behaviors than individual professional values.

In the present study, the interaction term between professional values and perceived ethical climate was found to significantly and negatively predict dignity‐protecting behaviors, providing empirical support for H_3b_ over H_3a_. These two directional hypotheses had been derived a priori from two competing theoretical perspectives, trait activation theory and situational strength theory, precisely because the existing literature offered no clear basis for privileging one direction over the other. Simple slope analyses revealed that the effect of professional values on dignity‐protecting behaviors remained strong and significant under conditions of low perceived ethical climate (effect = 0.203, *p* < 0.001), weakened at mean levels (effect = 0.093, *p* = 0.001), and lost statistical significance entirely at high levels (effect = 0.017, *p* = 0.608). This pattern is inconsistent with the direction predicted by trait activation theory, which anticipates that strong and consistent situational cues should strengthen the translation of a relevant individual trait into behavior [33], and instead corresponds to the direction predicted by situational strength theory, which proposes that individual differences may lose their determinacy over behavior under strong and clear situational conditions [34]. According to this theory, strong situations that present clear and consistent expectations suppress behavioral differences among individuals, thereby homogenizing behavior, whereas ambiguous and weak situations allow individual characteristics to become more determinative of behavior. The nursing ethics literature also supports this mechanism. Groothuizen (2024) argues that during nurses’ socialization into the clinical practice environment, the determinacy of individual moral values over behavior may weaken under conditions in which the norms prevailing in the unit are strong and consistent [49]. Similarly, Copeland (2019), who approaches moral action in nursing ethics from an ecological perspective, argues that an individual nurse’s moral awareness and action are situated within multiple nested systems—including unit culture—and that moral behavior therefore cannot be explained by individual values alone [50].

ICUs present a particularly noteworthy context within this theoretical framework. Life‐threatening clinical presentations, the intensity of ethical dilemmas, and the limited scope of patient autonomy [46] render ICUs strong situations in which behavioral expectations are exceptionally clear, consistent, and compelling. From this perspective, the pattern observed in the present study can be interpreted as follows: When nurses perceive the ethical climate of their work environment as strong, dignity‐protecting behavior may become more strongly guided by clear and consistent contextual expectations and therefore less dependent on individual professional values. Conversely, when ethical climate is perceived as weak, the absence of clear contextual guidance may increase nurses’ reliance on their internalized professional values to sustain dignity‐protecting behavior; professional values may thus function as a compensatory resource under these conditions. While the direction of this moderating effect was predicted a priori by H_3b_, the specific mechanism proposed above, namely that strong contextual expectations may reduce reliance on individual professional values under a strongly perceived ethical climate, was formulated with reference to the observed pattern; it therefore warrants direct empirical testing in future research. Nevertheless, given the nature of the cross‐sectional design, a causal interpretation of these relationships is not possible; these results warrant testing through longitudinal studies.

### 5.1. Limitations

This study has several limitations that should be considered when interpreting the results. First, the cross‐sectional design does not allow for causal inference. Longitudinal or experimental designs are needed to clarify the temporal and causal ordering of these relationships. Second, all data were collected from the same participants at a single time point using self‐report methods, which raises the possibility of common method bias. Harman’s single‐factor test indicated that the first unrotated factor accounted for 40.63% of the total variance across all 74 scale items. However, Harman’s single‐factor test alone cannot rule out the possibility of common method bias. Third, the sample was drawn from the adult ICUs of six private hospitals affiliated with a single foundation in Istanbul, which shared a common management structure and nursing care standards. Although this institutional homogeneity was advantageous for isolating the study variables, it may itself have contributed to a relatively uniform ethical climate across units, and it clearly limits the generalizability of the results to public hospitals, university hospitals, or institutions with more heterogeneous organizational cultures, as well as to different geographic and cultural contexts. Fourth, the reliability coefficients obtained in this sample were notably high; given the length of the scales and relatively high mean inter‐item correlations observed, some item redundancy cannot be ruled out. Future studies could examine item‐level psychometric properties in more detail, for example, using confirmatory factor analysis or item response theory approaches. Fifth, although ethical climate is often discussed as a shared, unit‐level phenomenon, it was measured and analyzed at the individual level in this study. Hospital‐level identifiers were not retained in the dataset, which precluded aggregating individual scores to the unit level; future studies should record institutional membership to enable such aggregation and to formally assess within‐unit agreement before treating ethical climate as a shared organizational construct. Sixth, although the direction of the moderating effect was predicted a priori, the specific substitution mechanism proposed to explain why this pattern occurs was formulated with reference to the observed data rather than specified in advance and has not itself been directly tested. Finally, the quantitative design did not capture nurses’ subjective experience of how ethical climate shapes the translation of professional values into behavior. Qualitative or mixed‐methods research is needed to gain deeper insight into the mechanism underlying this moderation pattern observed in this study.

## 6. Conclusion

The study results revealed that both professional values and perceived ethical climate significantly and positively predicted dignity‐protecting behaviors, with large effect sizes. Furthermore, perceived ethical climate was found to significantly moderate the relationship between professional values and dignity‐protecting behaviors. This moderating effect exhibited a pattern in which the effect of professional values weakened and lost statistical significance under conditions of a strong ethical climate, whereas it remained strong under conditions of a weak ethical climate.

### 6.1. Implications for Nursing Management

Interventions aimed at strengthening dignity‐protecting behaviors in ICUs should not be limited to educational programs targeting nurses’ individual professional values alone; priority should also be given to organizational arrangements designed to foster a supportive ethical work environment. Because professional values showed a stronger association with dignity‐protecting behavior when ethical climate was perceived as weak, managerial interventions aimed at fostering a supportive ethical work environment should be accompanied by efforts to reinforce nurses’ professional values. Management is encouraged to develop concrete policies that strengthen transparent decision‐making processes, sensitivity to patient rights, and a climate conducive to ethical discussion within the team.

Undergraduate and in‐service education programs should emphasize that professional values function in interaction with the organizational context, and curricula should incorporate content aimed at developing individual moral resilience and ethical reasoning skills that enable nurses to sustain dignity‐protecting behavior even under conditions of a weak ethical climate. Given the cross‐sectional design of this study, longitudinal research is recommended to determine the causal direction of the relationships identified. Furthermore, qualitative or mixed‐methods studies are needed to gain deeper insight into the mechanisms underlying the moderation pattern observed. Finally, to enhance the generalizability of the results, these relationships should be re‐examined in samples drawn from different institutional cultures, hospital types, and geographic contexts.

## Funding

No funding was received for this manuscript.

## Conflicts of Interest

The authors declare no conflicts of interest.

## Data Availability

The data that support the findings of this study are available from the corresponding author upon reasonable request. The data are not publicly available due to privacy and ethical restrictions related to participant confidentiality.
